# Association of polymorphisms in ADAMTS-7 gene with the susceptibility to coronary artery disease - a systematic review and meta-analysis

**DOI:** 10.18632/aging.104118

**Published:** 2020-10-29

**Authors:** Davood K. Hosseini, Sharareh Ataikia, Hanieh K. Hosseini, Baoai Han, Haiying Sun

**Affiliations:** 1 Department of Medicine, Stanford University School of Medicine, Stanford, CA 94305, USA; 2 Department of Otolaryngology-Head and Neck Surgery, Stanford University School of Medicine, Stanford, CA 94305, USA; 3 Shahid Beheshti University School of Medicine, Tehran, Iran; 4 Department of Public Laboratory, Tianjin Medical University Cancer Institute and Hospital, National Clinical Research Center for Cancer, Tianjin, China; 5 Department of Otorhinolaryngology, Union Hospital, Tongji Medical College, Huazhong University of Science and Technology, Wuhan, Hubei, China

**Keywords:** coronary artery disease, ADAMTS7 polymorphism, coronary angiography, efferocytosis

## Abstract

Objective: To systematically review literature evidence to discover the association of ADAMTS7 (A Disintegrin And Metalloproteinase with Thrombospondin-like motifs 7) polymorphisms and the risk of developing CAD (coronary artery disease).

Data sources: A related literature search in online databases, including EMBASE, PubMed, and Web of Science was undertaken. The period covered was from 2007 to September 10, 2019.

Results: Of 256 citations retrieved, nine relevant studies were selected for detailed evaluation. Five SNPs (rs3825807, rs1994016, rs4380028, rs79265682, and rs28455815) in ADAMTS7 gene were identified among included studies. There were 51,851 cases and 89,998 controls included in four studies for SNP rs3825807, 13,403 cases and 11,381 controls included in two studies for SNP rs1994016, 37,838 cases and 38,245 controls included in two studies for SNP rs4380028, 3,133 cases and 5,423 controls included in one study for SNP rs79265682, 103,494 cases and 198,684 controls included in one study for SNP rs28455815. We found most consistent evidence for an association with CAD on coronary angiogram with ADAMTS7 SNP rs3825807 risk allele A in contrast to control G allele, followed by rs4380028 (C vs. T allele), and rs1994016 (C vs. T allele).

Conclusions: ADAMTS7 polymorphism is likely an important risk factor for development of CAD. Our data also suggest that the ADAMTS7 polymorphism may be a risk factor for CAD progression in patients who already have pathology in their coronary arteries.

Review methods: We included all studies in English language that reported correlation between the ADAMTS7 polymorphism and CAD in human cases.

## INTRODUCTION

Coronary artery disease (CAD), which is characterized by atherosclerosis and narrowing in coronary arteries is a major cause of mortality and morbidity worldwide. Despite reduced CAD mortality over the last decade in developed countries, it is still responsible for about one-third of all deaths worldwide in people aged 35 and older [1–3].

**Figure 1:**
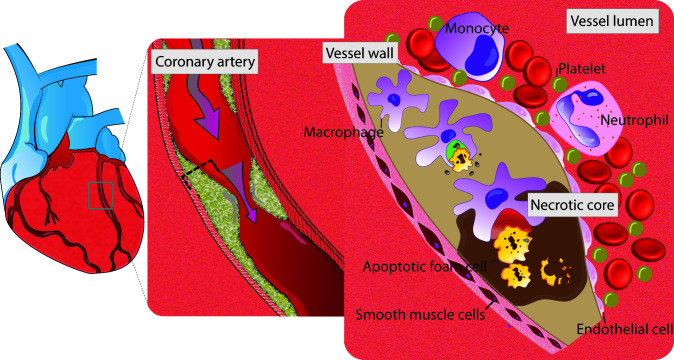
**Schematic view of efferocytosis (imbalance between red vs. green area).** Red zone, area with defective efferocytosis. Green zone, area with normal efferocytosis. Apoptotic cells in the growing atherosclerotic plaque are not recognized for efficient phagocytic clearance by macrophages. Impaired efferocytosis contributes to development of atherosclerosis. As a result, foam cells accumulate and the lesion expand with subsequent secondary necrosis in the apoptotic tissue. the end product of this cascade is vascular inflammation and instability of the lesion.

It is known that macrophages play an important role, in orchestra with smooth muscles cells (SMCs) and endothelial cells (EMCs) during inflammatory response that leads to atherosclerotic plaque formation. This includes the secretion of matrix metalloproteinases (MMPs) to extracellular space, and initiation of infiltrating and proliferative responses. More recent studies have revealed that impairment in “efferocytosis” - a process responsible for clearance of debris and remnant of inflammatory response - also results in initiation of inflammatory and proliferative circuits and subsequently plaque formation (Figure 1) [4, 5].

The complex pathophysiology underlying CAD depends on numerous environment and lifestyle risk factors of its individuals that is investigated through several studies which provide critical information regarding objectives for the primary and secondary preventive measures [2]. However, in practice, there are many patients who are diagnosed with CAD without clear explanation for development and progression of their premature coronary artery involvement into life threatening conditions by traditional risk factors. This arises the question of, what role do genetic components play in the acceleration of this disease.

Since the first Genome-Wide Association Studies (GWAS) conducted for CAD in 2007 [6–8], multiple studies with progressively larger sample sizes identified several genome-wide significant genetic loci associated with CAD [6, 9–12] among different patient populations.

More recently, the efforts to identify additional loci associated with CAD have revealed the responsibility of genetic factors for a significant percentage of the inter-individual variation in CAD and its progression. This necessitates a meta-analysis to combine the growing evidence to provide a comprehensive understanding of the genetic architecture of CAD, and enable precision medicine approaches by identifying subgroups of patients at increased risk of developing CAD or its complications.

One group of genetic factors that falls into this category is the ADAMTS7 (A Disintegrin And Metalloproteinase with Thrombospondin-like motifs 7), located on chromosome 15: 78,759,203-78,811,431 and has 5 transcripts (splice variants) that code for a proteolytic enzyme-ADAMTS7, which produced and secreted by macrophages, and contains 1686 amino acids. Following activation (through cleavage of the prodomain), the secreted enzyme can bind to cartilage oligomeric matrix protein (COMP), progranulin, and alpha2-macroglobulin [13, 14] and act as a proteolytic enzyme degrading the extracellular matrix of vessel walls including vascular extracellular matrix protein thrombospondin-5 (TSP5). Studies have previously shown that ADAMTS7 cleavage of TSP5 in animal models, facilitates vascular smooth muscle cell migration and promotes neointimal formation which leads to CAD [14–17].

Thus, the need arises to evaluate the genome data and determine the relationship between ADAMTS7 locus and coronary artery atherosclerotic vascular disease, specifically whether ADAMTS7 polymorphisms are linked to CAD or not.

Few human-case studies have showed that single nucleotide polymorphism in the ADAMTS7 gene is potentially responsible for tendency toward development of CAD. However, the results of these studies had not been consistently analyzed together among different patient populations.

Here, a meta-analysis was executed based on all eligible studies to assess the association of ADAMTS7 polymorphism with susceptibility to CAD, and to investigate the effects of ADAMTS7 polymorphism on progression of the disease in patients.

## RESULTS

### Study characteristics

**Figure 2:**
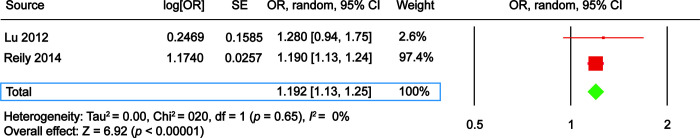
**The Forest plot comparing ADAMTS7 single nucleotide polymorphism (SNP) rs3825807 risk allele A vs. control allele G in development of coronary artery disease.** EmCAB Cohort, Emory Cardiovascular Biobank. SAS, Southampton Atherosclerosis Study; CI, confidence interval; OR, odds ratio; SE, standard error.

**Figure 3:**
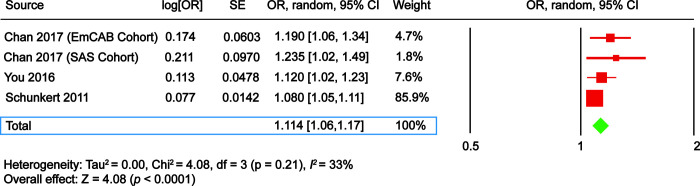
**The Forest plot comparing ADAMTS7 single nucleotide polymorphism (SNP) rs1994016 risk allele C vs. control allele T in development of coronary artery disease.** CI, confidence interval; OR, odds ratio; SE, standard error.

Our search terms produced 161 results on EMBASE, 30 results on PubMed, and 65 results on Web of Science, for a total of 256 records. Hand search of reference lists of articles identified an additional two studies [9, 18]. After excluding the duplicates (71), 187 articles were retained for initial screening by the title and abstract, yielding 61 that were assessed by full-text review for eligibility. Nine articles met inclusion criteria, and 52 studies were excluded. Among the excluded publications, 47 studies found to be irrelevant. Also, five publications were excluded because their full texts were not available. The data was examined separately for those studies with development of CAD and studies who reported the survival rate in patients who already have CAD (Figures 2 and 3).

### Coronary artery disease development

Our review included nine studies published between 2011 and 2019 and incorporated data collected from different countries (Table 1). Five SNPs (rs3825807, rs1994016, rs4380028, rs79265682, and rs28455815) in ADAMTS7 gene were identified among included studies. There were 51,851 cases and 89,998 controls included in 4 studies for SNP rs3825807, 13,403 cases and 11,381 controls included in 2 studies for SNP rs1994016, 37,838 cases and 38,245 controls included in 2 studies for SNP rs4380028, 3,133 cases and 5,423 controls included in one study for SNP rs79265682, 103,494 cases and 198,684 controls included in one study for SNP rs28455815. The gathered evidence suggests that there may be a close relationship between the SNP in the ADAMTS7 gene and CAD development.

**Table 1:** Summary of included studies.

	**Study**	**SNP**	**Risk allele/other allele**	**CAD**	**Country/ethnicity**	**No. of cases**	**No. of controls**	**Mean age (years)**	**Gender (M%)**
1	Schunkert (2011)	rs3825807	A/G	C.A. & clinical verification	EU Ancestry	45063	84589		51.03%
2	Reilly (2011)	rs1994016	C/T	C.A.	EU Ancestry	12393	7383	54.7	
	Peden (2011)	rs4380028	C/T	C.A.	EU Ancestry and South Asian	36828	34247	59.3	52.40%
3	Lu (2012)	rs1994016	C/T	C.A.	China	1010	3998	52.6	54.30%
		rs3825807	A/G	C.A.					
		rs4380028	C/T	C.A.					
4	Assimes (2016)	rs79265862	G/A	C.A.	China	3133	5423	65.5	59.05%
5	You (2016)	rs3825807	A/G	C.A.	China	4002	4152	60.3	66.20%
6	Chan (2017)	rs3825807	A/G	C.A.	UK	1076	283	62.5	70.10%
		rs3825807	A/G	C.A.	USA	1710	974	63.8	68.70%
7	Matsunaga (2019)	rs28455815	T/C	C.A. & clinical verification	Japan (BBJ-ToMMo, IMM, JPHC)	12494	28879	N/A	51.80%
					Japan (OACIS)	2808	7261	63.5	63.50%
					UK Ancestry	88192	162544		

Abbreviations: BBJ, Biobank Japan; CA, coronary angiography; EU, European Union; IMM, Iwate Tohoku Medical Megabank Organization; JPHC, Japan Public Health Center-based Prospective; OACIS, Osaka Acute Coronary Insufficiency Study; SNP, single nucleotide polymorphism; ToMMo, Tohoku Medical Megabank Organization; UK, United Kingdom; USA, United States of America.

### rs3825807

You et al. [19] conducted a study on two populations in China (Shijiazhuang and Wuhan). Their study revealed significant association between polymorphism in ADAMTS7 rs3825807 and development of CAD (OR 1.15; 95% CI 1.05-1.26).

Chan et al. [20] conducted two cohort studies (UK:EmCAB, and USA:SAS) and their data revealed that A allele is associated with susceptibility of CAD development among both Southampton atherosclerosis study and Emory university cohorts (OR 1.19; 95% CI 1.05-1.35, OR 1.23; 95% CI 1.02-1.48, respectively).

Schunkert et al. [9] reported a significant association between rs3825807 and CAD among their study cohorts form European descent (22,233 cases and 64,762 controls, followed by genotyping of and additional 60,738 subjects, with approximately half cases and half controls).

### rs1994016

Reilly et al. [21] carried out a case series study among patients with (case, n = 12,393) and without (control, n = 7,383) angiographic CAD, and reported significant association between ADAMTS7 SNP rs1994016, and angiographic CAD. However, they were unable to identify any association between ADAMTS7 SNP rs1994016, and myocardial infarction (MI), suggesting segregate genetic control for development of CAD and processes leading to full blown MI in the presence of CAD.

### rs4380028

Lu et al. [22] conducted 2 GWA studies among the Chinese Han descent, comparing 16,975 patients with CAD and 16,491 controls. Their study revealed a significant association of C/T polymorphism in rs4380028 and CAD. The pooled ORs (95% CI) were 1.17 (1.08-1.26, p <0.001). In this study they were unable to determine any association between CAD and other alleles that they were examined (rs1994016, rs3825807).

Peden et al. [23] studied a cohort among 36,828 CAD patients and 34,247 controls, and their data was significant with association of C/T polymorphism in rs4380028 and CAD with ORs (95% CI) of 1.07(1.05, 1.09).

### rs28455815

Matsunaga et al. [18] studied three population groups in their study, including two Japanese populations and one group with UK ancestry. Their data revealed significant association between T allele in ADAMTS7 and CAD (first group of Japanese population: OR of 1.12 (1.07-1.16), second group of Japanese: OR of 1.12 (1.04-1.20), and UK ancestry group: OR of 1.12 (1.08-1.16)).

### rs79265682

In 2016 Assimes et al. [24] reported significant association between GG allele in rs79265682 and CAD with OR (96%CI) of 1.67 (1.64-1.70).

### Coronary artery disease outcome

Pereira et al. [25] studied a cohort of 1,128 patients with CAD (confirmed with angiography), were genotyped for rs3825807 A/G. Prospective long term follow-up (mean 5.3 years) revealed that the AA genotype was a significant independent risk factor for mortality due to cardiovascular cause compared to the reference genotype GG (hazard ratio = 2.7).

Their data revealed that at the end of the follow-up (mean 5.3 years), the estimated survival was 89.8%, 82.2%, and 72.3% for GG genotype, AG, and AA genotype respectively after about 70 months and remained for about 168 months.

## DISCUSSION

To our knowledge, this is the first universal systematic review of the worldwide literature based on electronic database searches to assess different aspects of genetic variation in ADAMTS7 gene and its association with CAD. We found promising outcome data favoring for the importance of genetic evaluation in patients with preexisting risk factors and/or patient with history of CAD without traditional CAD risk factors.

We assessed nine studies that investigated the relationship between ADAMTS7 polymorphism and the presence of CAD. These trials were conducted in different countries. Our data reveals that ADAMTS7 was consistently related to CAD identified by coronary angiography. Moreover, the outcome of CAD in the patient with angiographic proven CAD, in the long-term follow up, confirmed the influence of ADAMTS7 polymorphism on CAD. These findings suggest that genetic assessment should be considered, both in the development of CAD and also in patients who are already suffering from it, to get a better understanding of coronary artery involvement as a major cause of mortality, especially when familial history suggest the possibility of genetic involvement.

In the other studies, the interaction of genes and environment have produced an interesting observation that allelic variation at rs7178051 that are associated with reduced ADAMTS7 expression confers CAD protection, which is stronger in never-smokers than in cigarette smokers [26, 27]. Enhanced vascular ADAMTS7 expression may lead to the loss of protection from CAD, leading to the suggestion that inhibition of ADAMTS7 may be especially helpful for individuals who smoke cigarettes. In addition, it has been shown that SNPs variants has a role in development of other vascular diseases, for example Chen et al. described the associated between the rs3825807 variants with development of ischemic stroke [28].

Our review has weaknesses inherent to systematic review approaches. There are several members of the ADAMTS that identified, however the relevant data regarding the role of the other member of ADAMTS and their effect on the development of the coronary artery disease are limited, and further studies required. Our review includes few studies, which limits the strength of conclusions that can be drawn. Further studies, warranted to confirm our findings in larger patient population and wider geographic area. Furthermore, it is ripe for research to see how the genetic changes in ADAMTS7 would be translated into changes in plasma level of ADAMTS7 in patients with CAD.

## MATERIALS AND METHODS

### Search strategy

The systematic review of the literature and meta-analysis were developed and conducted according to the PRISMA (Preferred Reporting Items for Systematic Reviews and Meta-Analysis) guidelines [29]. Our study objective was to summarize the genetic SNP missense mutations in ADAMTS7 in the literature and to investigate its relationship to the presence, progression and implication for CAD.

**Figure 4:**
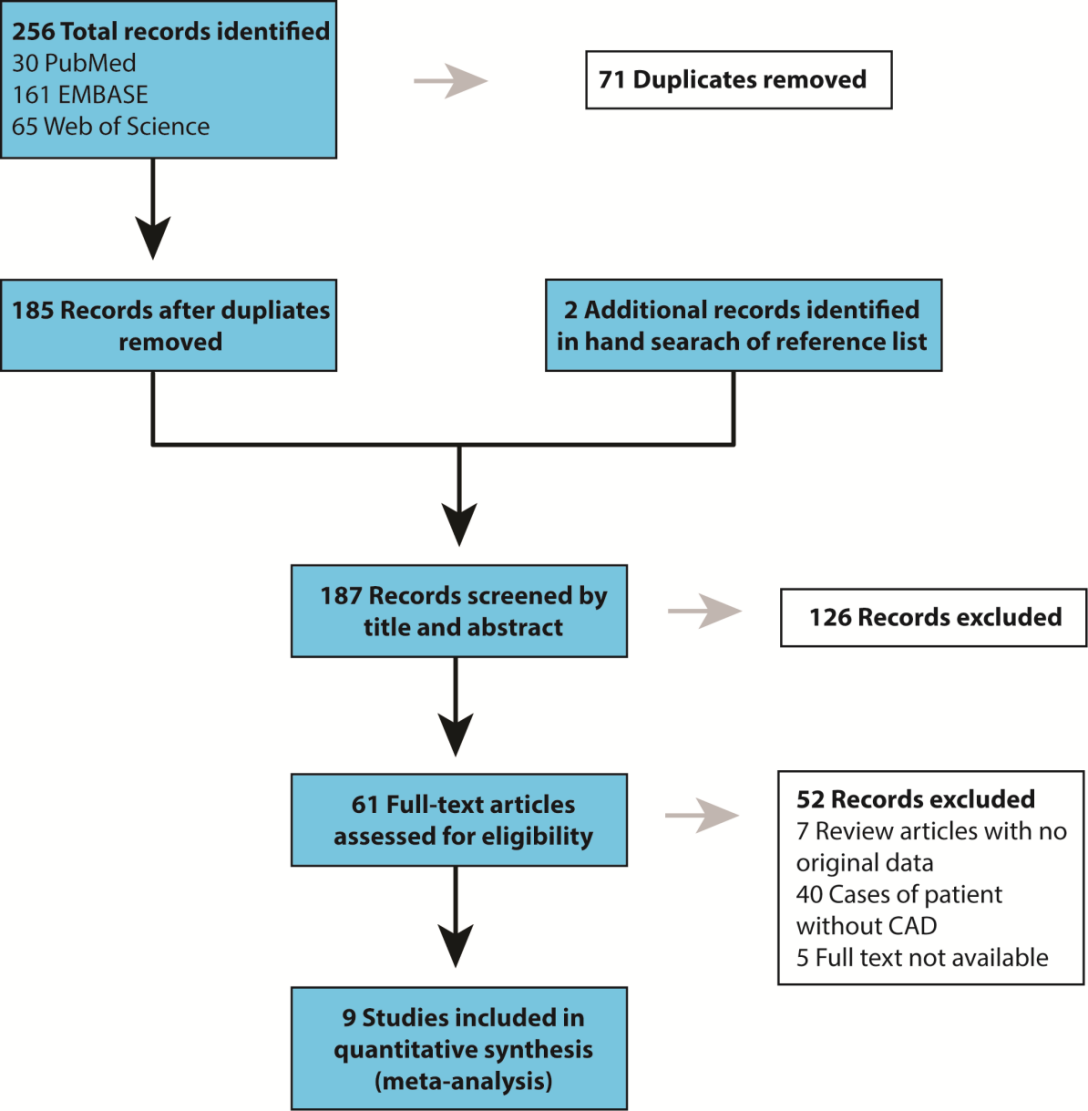
**PRISMA flowchart of article search in PubMed, EMBASE, and Web of Science and study selection.** CAD, coronary artery disease.

A related literature search in online databases, including EMBASE, PubMed, and Web of Science were undertaken. The following search terms were used: ("ADAMTS7 Protein" [Mesh] OR adamts7 [tw] OR “adamts 7” [tw] AND English [lang]) for the period of 2007 to September 10, 2019. Search results were limited to human studies and also in the English language. Review articles with data on ADAMTS7 were also checked, in order to identify studies that were missed by our database search. In addition, we reviewed articles unavailable online by obtaining hard copy scans via Stanford Lane Medical Library’s interlibrary loan and document delivery service. The detailed steps of the literature review are shown in Figure 4.

### Inclusion Criteria and Study Selection

Published studies were considered eligible for inclusion based on the following criteria: 1. an original study investigating ADAMTS7 SNP associated with CAD, 2. a human study, 3. published in English, 4. Coronary stenosis defined as > 50% narrowing in coronary angiographic exam, 5. data regarding CAD outcomes. Selected studies were divided according to whether the data was related to CAD development, or survival rate in patients with CAD. The exclusion criteria for our study were: 1. duplicated studies, 2. review articles without original data on ADAMTS7 SNP associated with CAD, 3, letters and case reports, 4. studies without gene polymorphism, 5. Unavailable data and abstracts from unpublished studies. Additional studies identified by reviewing the reference lists of the retrieved literature were likewise manually checked using the same inclusion and exclusion criteria.

Two authors (S.A. and H.S.) independently assessed the eligibility of studies, with any discrepancies settled by consensus with a third author (D.K.H). We excluded duplicate studies using Zetero software prior to screening. Initial screening was done with the article abstract and title to generate a list of potentially relevant studies. Full-text review was undertaken to determine eligibility by our inclusion criteria.

### Data extraction strategy and assessment of study quality

The following data was extracted from the included studies by two authors (S.A. and H.S.): 1. basic information: first author, publication year, sample size of case (patients with CAD) and control groups (patients without CAD), 2. demographic data: mean age, male/female ratio, country and ethnicity of cases and controls, 3. method of coronary artery evaluation, genotyping, genotype distributions, and relevant SNP polymorphism. Newcastle-Ottawa scale (NOS) was used to assess the quality of each included study. Studies with a score greater than 7 were considered as high-quality studies. Disagreements were resolved by consensus with a third author (D.K.H).

### Statistical methods

All analyses were conducted using CMA (Comprehensive Meta-analysis) Software (Version 3) [30]. From each study, the title, sample size, mean and standard deviation, were entered using the standard mean difference method. Odds ratios (ORs) and hazardous ratios (HRs) with corresponding 95% CIs were used to evaluate the clinicopathologic and prognostic value. HR < 1 with 95% CI not overlapping 1 was indicated a better survival. Cochrane *Q* statistic, *I^2^* statistic and *Tau-squared* were calculated to assess heterogeneity. Given that the various heterogeneities among individual ORs and HRs, the random effects model was utilized to generate summary statistics. *P*-value < 0.05 was considered to be statistically significant unless otherwise specified.
